# TMBIM6, a potential virus target protein identified by integrated multiomics data analysis in SARS-CoV-2-infected host cells

**DOI:** 10.18632/aging.202718

**Published:** 2021-03-19

**Authors:** Qizheng Han, Junhao Wang, Haihua Luo, Lei Li, Xinya Lu, Aihua Liu, Yongqiang Deng, Yong Jiang

**Affiliations:** 1 Guangdong Provincial Key Laboratory of Proteomics, State Key Laboratory of Organ Failure Research, School of Basic Medical Sciences, Southern Medical University, Guangzhou, China; 2 Department of Respiratory and Critical Care Medicine, Nanfang Hospital, Southern Medical University, Guangzhou, China

**Keywords:** SARS-CoV-2, COVID-19, TMBIM6, apoptosis, multiomics data, bioinformatics

## Abstract

Coronavirus disease 2019 (COVID-19) is caused by severe acute respiratory syndrome coronavirus 2 (SARS-CoV-2). In this study, we collected open access data to analyze the mechanisms associated with SARS-CoV-2 infection. Gene set enrichment analysis (GSEA) revealed that apoptosis-related pathways were enriched in the cells after SARS-CoV-2 infection, and the results of differential expression analysis showed that biological functions related to endoplasmic reticulum stress (ERS) and lipid metabolism were disordered. TMBIM6 was identified as a potential target for SARS-CoV-2 in host cells through weighted gene coexpression network analysis (WGCNA) of the time course of expression of host and viral proteins. The expression and related functions of TMBIM6 were subsequently analyzed to illuminate how viral proteins interfere with the physiological function of host cells. The potential function of viral proteins was further analyzed by GEne Network Inference with Ensemble of trees (GENIE3). This study identified TMBIM6 as a target protein associated with the pathogenesis of SARS-CoV-2, which might provide a novel therapeutic approach for COVID-19 in the future.

## INTRODUCTION

The outbreak of SARS-CoV-2 has disseminated to 216 countries and regions around the world [1]. The pneumonia patients infected by this novel coronavirus have clinical manifestations that include fever, fatigue and dry cough; approximately half of them develop dyspnea one week later, and some patients with severe disease progress rapidly to acute respiratory distress syndrome (ARDS), septic shock and metabolic acidosis [2, 3]. The 29,903-nucleotide genome of SARS-CoV-2 encodes 14 major open reading frames (ORFs), which can be further processed into 4 structural proteins, namely, the glycoprotein spike (S), envelope (E), nucleocapsid (N) and membrane (M), 16 nonstructural proteins (nsp1 to nsp16), and at least 8 accessory proteins (ORF3a, ORF6, ORF7a, ORF7b, ORF8, ORF9a, ORF9b and ORF10) [4, 5]. Similar to SARS-CoV [6, 7], the spike protein of SARS-CoV-2 directly interacts with the receptor ACE2 on the membrane of human airway epithelial cells to promote virus invasion [8, 9]. To date, many studies have focused on the receptors for the spike protein of SARS-CoV-2, especially ACE2; however, the intracellular mechanism used by the virus to achieve amplification and regulate the protein expression of host cells is largely unknown. Due to the rapid development of omic techniques, it is possible to systematically illuminate the mechanism underlying the cellular response to the virus. Recently, Bojkova et al. investigated protein expression in host cells infected with SARS-CoV-2 by translatome and proteome data analysis [10]; Emanuel et al. investigated the expression of genes in cells infected with SARS-CoV-2 by RNA sequencing (RNA seq) [11]. Previous studies have demonstrated that increased synthesis of viral proteins is a prerequisite for virus amplification in host cells, and the virus might disrupt or hijack the intracellular anti-virus mechanism to support viral propagation. Further efforts are needed to illustrate the molecular drivers of SARS-CoV-2 pathogenesis.

In this study, gene set enrichment analysis (GSEA) with proteome data was performed to analyze the enriched signaling pathways of differentially expressed proteins induced by SARS-CoV-2 virus invasion. Differential expression analysis was utilized to obtain host proteins regulated by SARS-CoV-2 at the posttranscriptional level. After weighted coexpression network analysis (WGCNA) of the proteome data, we identified a key protein, TMBIM6, that was highly coexpressed with the viral proteins. Moreover, the regulatory proteins associated with the viral proteins were predicted by GEne Network Inference with Ensemble of trees (GENIE3). In general, through integrated bioinformatic analysis of transcriptome, translatome and proteome data, we found that the TMBIM6 protein in host cells was inhibited by SARS-CoV-2 at the posttranscriptional level to mediate the host cell response to virus infection.

## RESULTS

### Open access data from human tissues and cells infected with SARS-CoV-2 virus

Proteome and translatome data from human Caco-2 cells infected with SARS-CoV-2 from Bojkova et al. were downloaded and used for subsequent analysis [10]. This data consisted of the quantification of 6,381 proteins in human Caco-2 cell secretomes at four time points after infection with SARS-CoV-2 virus. We also obtained transcriptome data of Caco-2 cells infected with SARS-CoV-2 at different time points from Emanuel et al., which consists of 45058 genes with quantifications in Caco-2 cells [11]. The transcriptome data of autopsied lungs from patients who died due to SARS-CoV-2 infection (GSE150316) and different virus-infected A549 cells [12] were used for subsequent verification. The proteome data from cells infected with SARS-CoV-2 [13], influenza A virus (IAV) [14] and respiratory syncytial virus (RSV) [15], which provided the information of differentially expressed proteins, were also collected as a source of circumstantial evidence.

### Host cellular response to SARS-CoV-2

**Figure 1:**
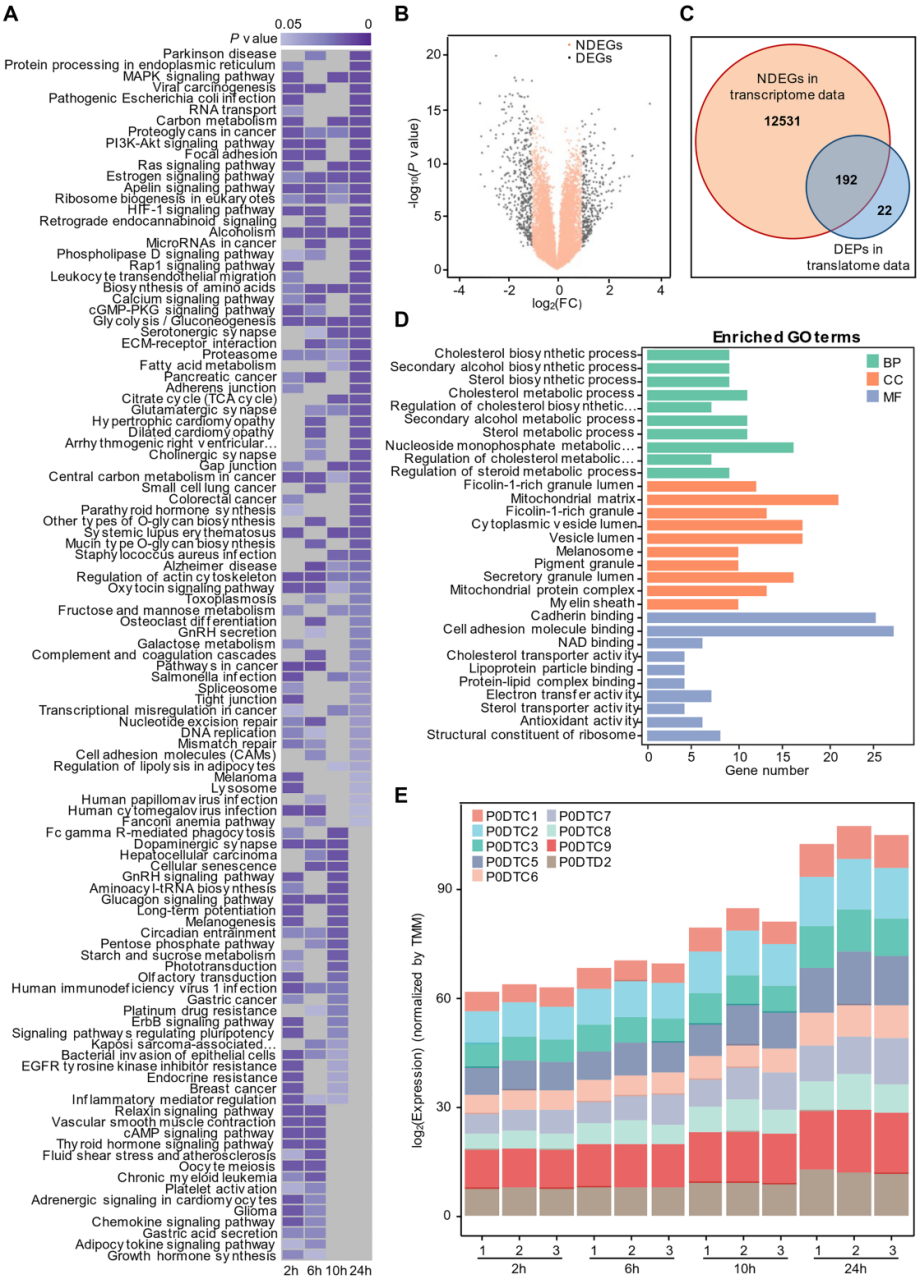
**Time-resolved multiomics profiling of the cellular response to SARS-COV-2 infection.** (**A**) Gene set enrichment analysis by time series. Significant KEGG terms (*P* value < 0.05, *n* = 3) are presented with a heatmap. Lower *P* values are shown in dark purple, and higher *P* values are light purple. Nonsignificant pathways are presented in gray. (**B**) Volcano plot of non-differentially expressed genes (NDEGs) from SARS-CoV-2-infected cells. The orange dots represent NDEGs with an absolute log_2_ (fold change) ≤ 1 or a *P* value ≥ 0.05 (*n* = 2) compared with the control. For the small sample number, the *P* value was calculated with Limma-voom R package following the method reported by Ritchie ME et al. [48]. (**C**) Venn diagram showing the overlap of non-differentially expressed genes (NDEGs) in the transcriptional data and differentially expressed proteins (DEPs) (*P* value < 0.05 and absolute log_2_ (fold change) > 1) in the translational data (*n* = 3). (**D**) Analysis of GO terms for the 192 proteins shared by NDEGs and DEPs with *P* value < 0.05. (BP: Biological Process; CC: Cellular Component; MF: Molecular Function) (**E**) The time-dependent expression of SARS-COV-2 proteins detected by proteomics.

With the proteome data, we first compared the protein expression in SARS-CoV-2-infected cells and mock-infected cells at different times. Through GSEA, we obtained the enrichment results of four time points separately on the proteome data with *P* value < 0.05. The results are then merged into one heatmap [16]. Because the influence of the early virus is little significant, we paid more attention to the influence of the virus at 24 hours. At the same time, in order to better display the results, we sorted the enrichment results at 24 hours according to the *P* value, and the enrichment results at other time points were displayed in the order of 24 hours. If there is no enrichment for a certain pathway at a certain time, we will display it with a grey module (Figure 1A). Consistent with the findings of Appelberg et al. [17], our results demonstrated that the ErbB, PI3K-AKT, HIF-1 and Rap1 signaling pathways were activated in Caco-2 cells infected with SARS-CoV-2. Interestingly, we also found that some metabolism-related signaling pathways, including protein processing in the endoplasmic reticulum (ER), carbon metabolism, ribosome biogenesis in eukaryotes, calcium signaling pathway and glycolysis/gluconeogenesis, were involved in the host cellular response to SARS-CoV-2 invasion.

### Identification of host proteins influenced by SARS-CoV-2

With transcriptome data, the R package of Limma-voom was used to analyze differentially expressed genes (DEGs), which were visualized by a volcano plot (Figure 1B), and differentially expressed proteins (DEPs) were defined by two-sided unpaired student’s t test with equal variance assumed according to the original author using translatome data. To understand SARS-CoV-2's influence on cells at the post-transcriptional level, and remove some of the stress responses made by host cells at the transcriptional level due to SARS-CoV-2's invasion, we performed intersection analysis to identify the overlap of proteins encoded by non-differentially expressed genes (NDEGs) and DEPs in Caco-2 cells infected with SARS-CoV-2 (Figure 1C). Interestingly, we found that most differentially expressed proteins are translated by non-differentially expressed genes. Further analysis of Gene Ontology (GO) enrichment showed that the 192 shared proteins might involve in several biological process, including mitochondria-related functions, cholesterol biosynthesis processes and protein transport and so on (Figure 1D). And Daniloski et al. [18] identified that SARS-CoV-2 infection negatively downregulates the cholesterol synthesis pathway and viral infection can be counteracted by drug treatments that upregulate the same pathway, which is consistent with our results. In parallel, we also performed intersection analysis to identify the overlap of proteins encoded by DEGs and DEPs in Caco-2 cells infected with SARS-CoV-2, and the results are displayed in (Supplementary Figure 1). The results indicating that host cells may influence the expression of related proteins, catabolic process, metabolic process and other related pathways through the regulation at the transcriptional level.

### Identification of SARS-CoV-2-related protein modules through WGCNA

**Figure 2:**
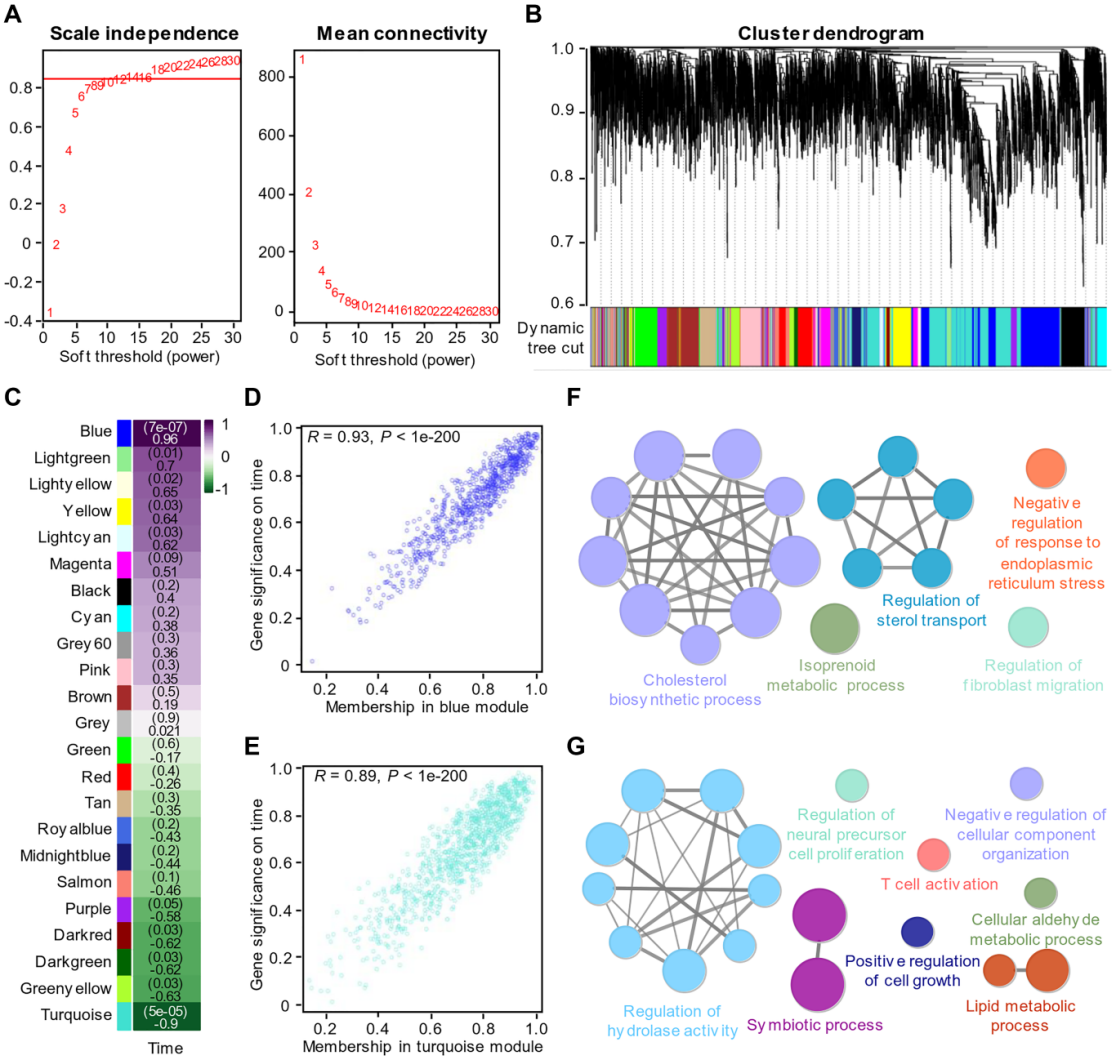
**Identification of key modules through WGCNA of the proteomics data.** (**A**) Analysis of the scale-free fit index (left) and the mean connectivity (right) for various soft-thresholding powers. (**B**) Clustered dendrogram of the top 5000 proteins based on the dissimilarity measure. (**C**) Heatmap of the correlations between modules and virus amplification over time. Each cell contains the correlation coefficient and *P* value. (**D**) The corresponding correlation and P value between proteins in the blue module and virus amplification over time. (**E**) The corresponding correlation and *P* value between proteins in the turquoise module and virus amplification over time. (**F**) Enrichment analysis of the proteins in the turquoise module with *P* value < 0.05. (**G**) Enrichment analysis of the proteins in the blue module with *P* value < 0.05.

In the article of Bojkova et al., we found that the morphology of host cells changed significantly 24 hours after infection, indicating that virus infection had a great impact on cells (Figure 1E). We therefore performed dimensional reduction with weighted gene co-expression network analysis, which is robust for small sample sizes, reduces the burden of multiple hypothesis testing, identifies clusters or networks of proteins that are potentially dysregulated, and analyzes the relationship between protein coexpression pattern and cell phenotype after virus infection. Also, several studies [19, 20] used WGCNA to analyze gene expression during virus infection. A total of twenty-three modules were identified by setting the soft-thresholding power as 8 (scale-free R² = 0.85) (Figure 2A), cut height as 0.25, minimal module size as 50 and threshold as 0.2 (Figure 2B). From the heatmap of module-trait correlation (Figure 2C), we found two modules, blue module and turquoise module, significantly correlated (*P* < 0.01) with virus amplification according to the corresponding correlation and *P* value (Figure 2D–2E). The expression of proteins in the module may be up-regulated or down-regulated. ClueGO was then used to reveal the potential biological processes of proteins in the blue and turquoise modules. The results demonstrated that the blue module proteins had a close relationship with cholesterol biosynthesis process, positive regulation of sterol transport and negative regulation of response to ERS (Figure 2G), which is also consistent with our previous findings, and the turquoise module proteins were closely related to the regulation of hydrolase activity, T cell activation and liquid metabolic process (Figure 2F).

### The regulatory relationship between SARS-CoV-2 proteins and host proteins

**Figure 3:**
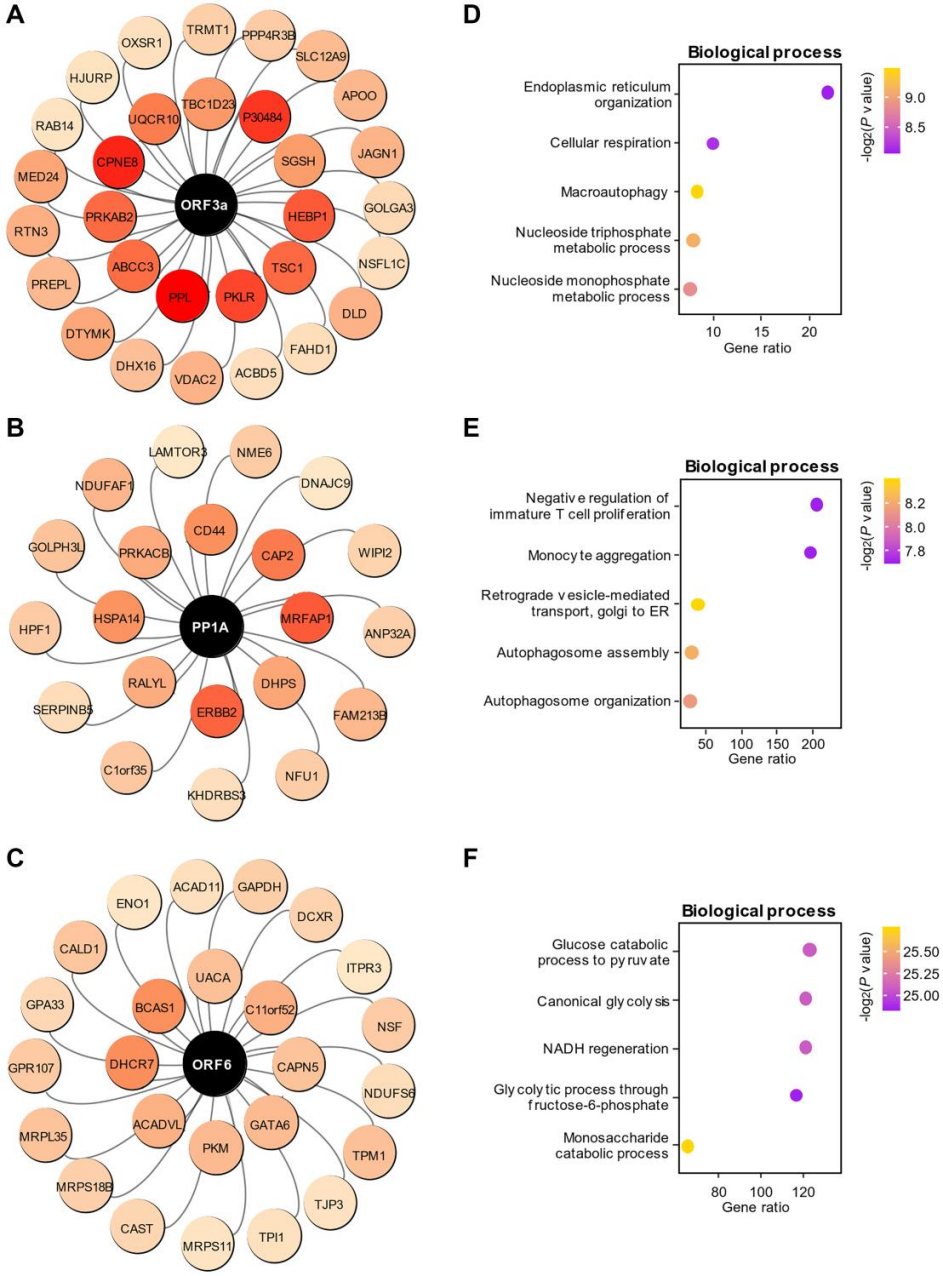
**Functional prediction of viral proteins in the blue module.** (**A–C**) The paired associations of the top three viral proteins, ORF3a (A), PP1A (B) and ORF6 (C), with high-weight host proteins in the regulation predicted by GENIE3. The color depth represents the weight of the pairing between viral proteins and each host protein. (**D–F**) Enrichment of biological processes associated with host proteins regulated by the viral proteins ORF3a (D), PP1A (E) and ORF6 (F).

In the WGCNA results, we noted that six viral proteins were clustered in the blue module, suggesting that those viral proteins play an important role in the amplification of SARS-CoV-2 in the cells. After analyzing the weights between the paired proteins with GENIE3, we obtained the close regulatory relationships between viral proteins and host proteins in the cells. The top 0.2% (77 pairs) of the 38281 related pairs were chosen for further analysis, and we found that these paired proteins were selectively associated with ORF3a (Figure 3A), PP1A (Figure 3B) and ORF6 (Figure 3C). Intriguingly, ORF3a had a higher weight in regulating host proteins than other viral proteins, indicating an important role of ORF3a in the replication of SARS-CoV-2 in the cells. GO enrichment analysis demonstrated that ORF3a, PP1A and ORF6 were functionally related to ER organization and macroautophagy (Figure 3D), autophagosome (Figure 3E) and glucose catabolic process (Figure 3F), respectively. Due to the relatively low weights of S, N and ORF9b for regulating host proteins, the top 30 host proteins regulated by these proteins were chosen for GO enrichment analysis (Supplementary Figure 2A–2C). GO enrichment analysis demonstrated that S, N and ORF9b were functionally related to metabolic pathway (Supplementary Figure 2D), inflammatory response (Supplementary Figure 2E) and cell cycle (Supplementary Figure 2F), respectively. The functional domains of the viral proteins are also displayed in (Supplementary Figure 3) to provide a preliminary understanding of the structure of SARS-CoV-2 viral proteins.

### Identification of hub proteins in the blue and turquoise modules

**Figure 4:**
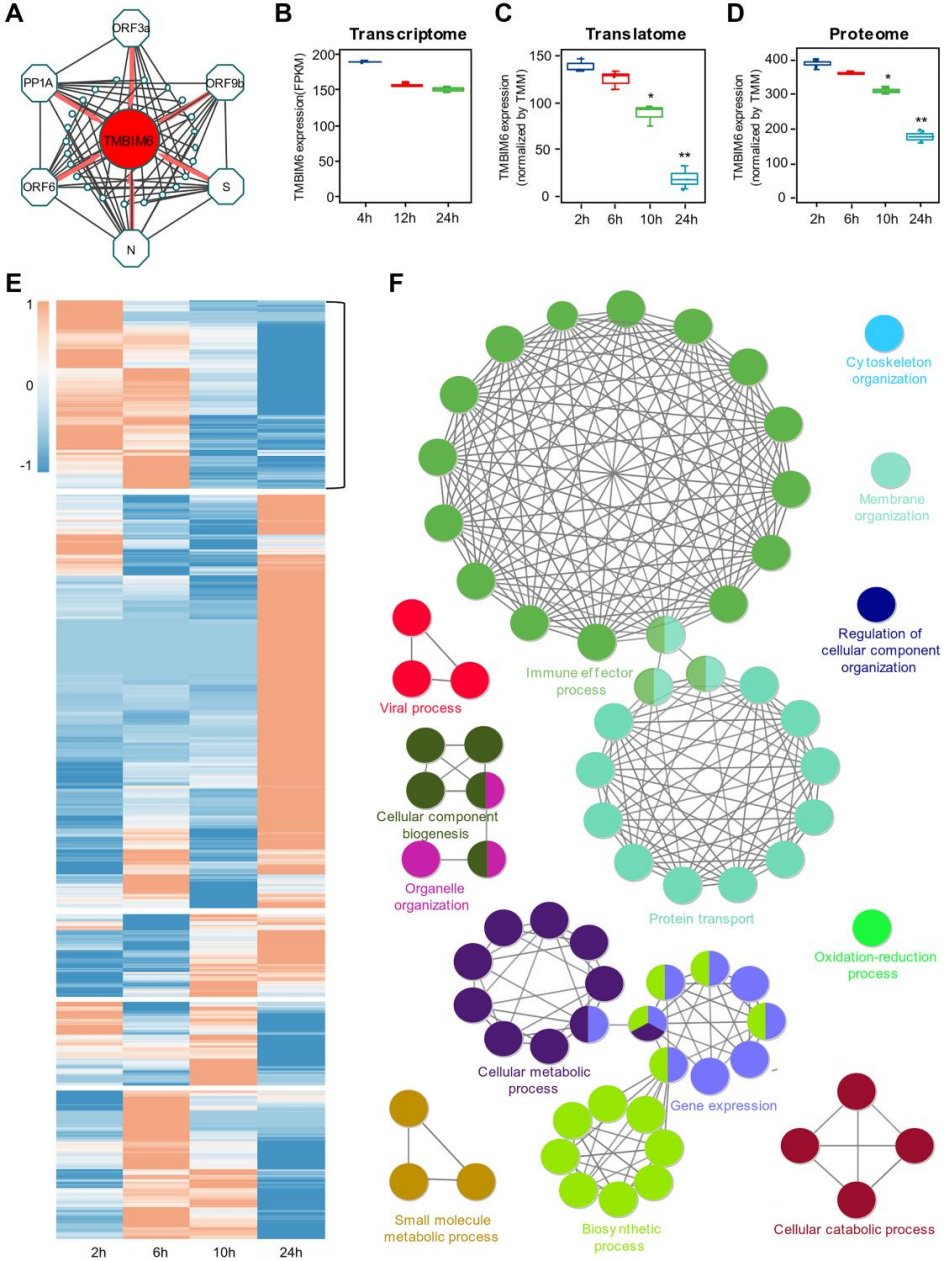
**Identification of the host protein TMBIM6 as a potential target for viral pathogenesis.** (**A**) Top 10 coexpression relationships of SARS-COV-2 proteins, including PP1A, S, ORF3a, ORF6, ORF9b and N, and host proteins from the blue module. TMBIM6 was found to be coexpressed with these six viral proteins. (**B–D**) The expression of TMBIM6 at different times based on multiomics data from SARS-CoV-2-infected cells. *n* = 3, ^*^P value < 0.05, ^**^P value < 0.001. (**E**) Heatmap of expression clustering for proteins from the translational data. The cluster with an expression pattern similar to TMBIM6 was selected for the next step of analysis. (**F**) Enrichment analysis of biological processes of proteins from the selected cluster with *P* value < 0.05.

Since SARS-CoV-2-related proteins were found to be mainly clustered in the blue module, we selected the top 10 host proteins coexpressed with each viral protein in the blue module, as shown in (Table 1). Interestingly, we found that TMBIM6 showed prominent coexpression with all viral proteins identified in the blue module, indicating that TMBIM6 is a key element influenced by the infection of SARS-CoV-2 in the cells (Figure 4A). Previous studies have reported that TMBIM6 acts as a BAX inhibitor by blocking BAX translocation from the ER to mitochondria [21, 22]. We found that BAX [23] was in the turquoise module, which was in contrast to the blue module and related to the cellular stress response. Thus, we identified TMBIM6 as a hub protein in the blue module associated with viral proliferation.

**Table 1:** SARS-CoV-2-related proteins and their corresponding top 10 coexpressed host proteins in blue module.

**SARS-CoV-2-related proteins**	**Corresponding top 10 coexpressed host proteins**
N	PP1A, KRT18, TMBIM6, ORF9b, TFRC, S, KRT8, RAB5C, SCD
ORF3a	TMBIM6, ORF9b, PP1A, KRT8, KRT18, RAB5C, TFRC, IPO7, MTHFD1L, EEF1B2
ORF9b	TMBIM6, SLC3A2, PP1A, IPO7, ORF6, APLP2, RAB5C, STAG1, KRT18, ZC3HAV1
PP1A	SPC25, LRP1, TFRC, TMBIM6, SELENOS, SLC3A2, TKT, CYP51A1, HLA-B, IDI2
ORF6	PP1A, SLC3A2, TMBIM6, HLA-B, IDI2, SELENOS, PGPEP1, SPC25, TFRC, CYP51A1
S	TMBIM6, PP1A, ORF9b, ANXA13, KRT18, RAB5C, TFRC, KRT8, ORF6, ECH1

### Multiomics data analysis of the expression of TMBIM6

To determine whether the regulation of TMBIM6 expression occurred at the posttranscriptional level, we performed systematic studies on the expression of TMBIM6 with publicly available transcriptome (Figure 4B), translatome (Figure 4C) and proteome (Figure 4D) data from Caco-2 cells infected with SARS-CoV-2. From the transcriptome data, we found that there was no significant difference in the expression of TMBIM6 in Caco-2 cells during infection with SARS-CoV-2 for 24 h at the transcriptional level (Figure 4B). However, there was a significant decrease in TMBIM6 at both the translatome and proteome levels in 24 h. To certify that this phenomenon was specifically induced by SARS-CoV-2, we performed expression analysis with translatome and proteome data from mock-infected Caco-2 cells as the control and found that there was no significant difference in the expression of TMBIM6 during the 24 h observation period (Supplementary Figure 4A–4C). The heatmap displayed clustered proteins with an expression pattern similar to that of TMBIM6 in the translatome data (Figure 4E). The results of GO enrichment analysis revealed that the proteins in this cluster were related to the viral process, immune effector and biosynthesis process (Figure 4F). Further enrichment analysis of the proteins coexpressed with TMBIM6 demonstrated that TMBIM6 might be related to metabolic processes and chaperone-mediated protein folding (Supplementary Figure 5).

### Expression of TMBIM6 in different human tissues and A549 cells infected with SARS-CoV-2

**Figure 5:**
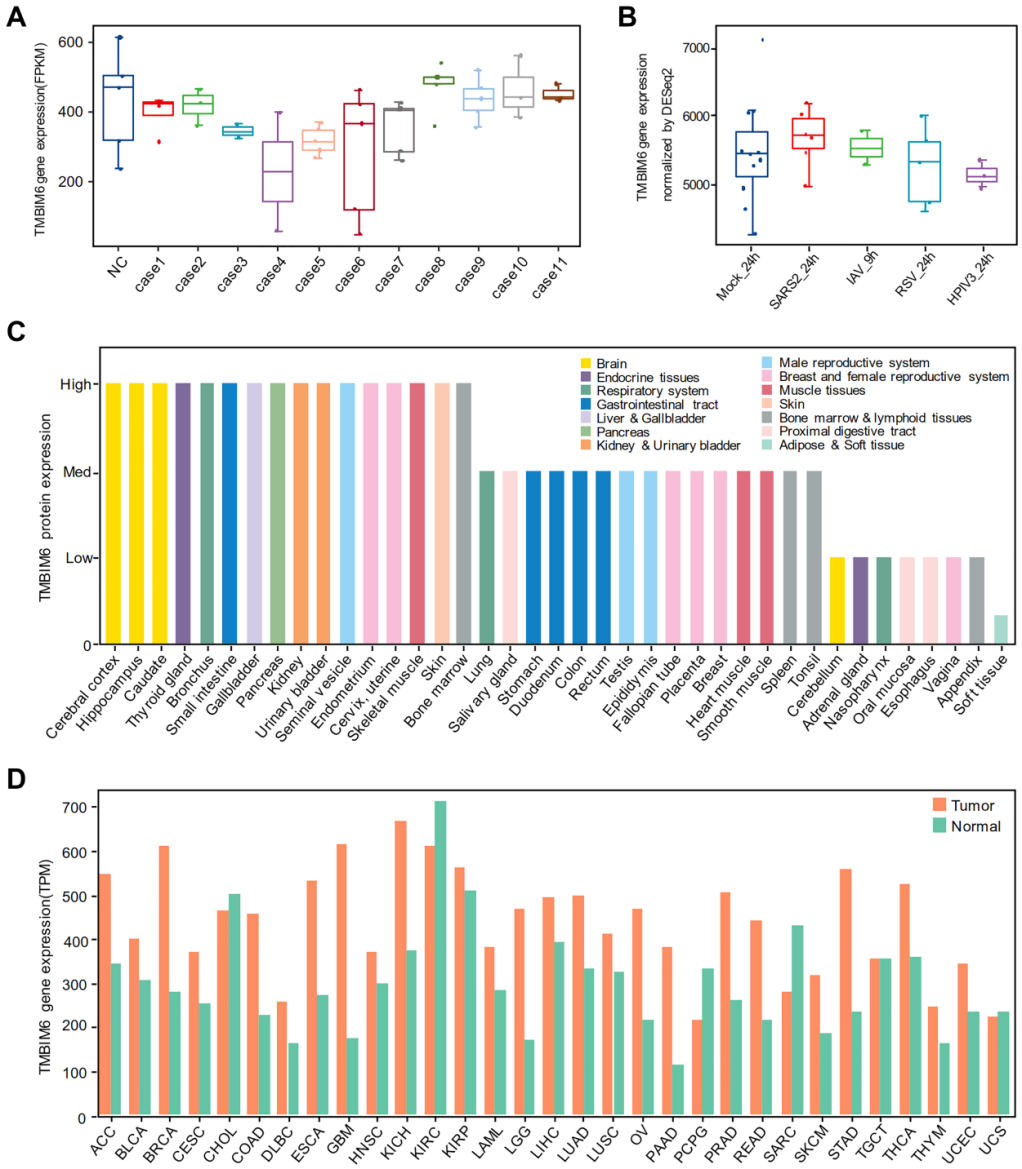
**The expression of TMBIM6 in different human tissues and A549 cells infected with different viruses.** (**A**) Nondifferential expression for TMBIM6 gene in autopsied lungs from patients who died from SARS-CoV-2 infection. (In all the cases, *P* value > 0.05, comparison to negative control). (**B**) Nondifferential expression for TMBIM6 gene in cells infected with different viruses at 24 h or 9 h. (*P* value > 0.05, comparison to mock control). (**C**) The protein expression of TMBIM6 in different human tissues from the HPA database. (**D**) The gene expression of TMBIM6 in tumor and normal tissues from the GEPIA2 database at the transcriptional level.

To verify the results above, we found another multiomics data to see the expression of TMBIM6. Analyzing the gene expression of TMBIM6 in autopsied lung tissues (Figure 5A) and we found that there was no significant difference in TMBIM6 expression at the transcriptional level. The results of the transcriptome analysis of cells invaded by different viruses showed that the gene expression of TMBIM6 was not significantly downregulated by SARS-CoV-2 or other viruses (Figure 5B). Moreover, we analysed Alexey Stukalov’s SARS-CoV-2-infected proteome data, which displays that TMBIM6 protein has a downward trend at the proteome level, as shown in the (Table 2). We also got differentially expressed proteins information of IAV infection from Coombs et al. (Supplementary Table 1) and that of RSV infection from Sande et al. (Supplementary Table 2), and both of them showed that TMBIM6 protein was not differentially expressed before and after infection with IAV and RSV.

**Table 2:** Statistical analysis of TMBIM6 protein changes at 3, 6, 12, 18, 24 and 30 h.

**Time points**	**Log2Foldchange**	***P* value**
3 h	–0.014587327	0.847830887
6 h	–0.046795833	0.767755994
12 h	–0.132258938	0.598284486
18 h	–0.727420294	0.064174941
24 h	–0.704769233	0.089075154
30 h	–0.706389819	0.149384731

To clarify the gene and protein expression of TMBIM6 in different human tissues, we performed expression analysis using the Human Protein Atlas (HPA) and Gene Expression Profiling Interactive Analysis (GEPIA2) and found that the TMBIM6 protein was highly expressed in most human tissues, including the brain, bronchus, kidney, small intestine, lung, and heart muscle (Figure 5C), and that the TMBIM6 gene was highly expressed in most human tissues at the transcriptional level, as well as tumors (Figure 5D).

## DISCUSSION

SARS-CoV-2 is currently the most harmful public health disaster. Previous studies have mainly focused on receptors for the spike protein of SARS-CoV-2, especially ACE2. In contrast, few studies have focused on multiomics data from cells infected with SARS-CoV-2. To date, the intracellular mechanism that is utilized by the virus to achieve replication in host cells is largely unknown. To gain insight into the activation of signaling pathways in SARS-CoV-2-infected cells, we acquired proteome data to perform GSEA. The GSEA results demonstrated that SARS-CoV-2 influenced the regulation of many signaling pathways, including the ErbB, PI3K-AKT, HIF-1 and Rap1 signaling pathways, in host cells, which was consistent with the findings of Appelberg et al. [17]. We also found that SARS-CoV-2 infection was involved in signaling pathways associated with metabolic processes, including glycolysis/gluconeogenesis, fatty acid metabolism and carbon metabolism, and protein processing in the ER. These results suggest that by utilizing intracellular mechanisms, SARS-CoV-2 completes virus replication, which leads to metabolic disorders and the ERS response in host cells.

To understand SARS-CoV-2's influence on cells at the post-transcriptional level, and remove some of the stress responses made by host cells at the transcriptional level due to SARS-CoV-2's invasion, we performed a combined analysis of proteins encoded by NDEGs and DEPs to identify the overlapping proteins in Caco-2 cells infected with SARS-CoV-2. The time-dependent expression of viral proteins represents virus replication in the cells; thus, we performed WGCNA on the proteome data to identify host proteins influenced by SARS-CoV-2. Intriguingly, the blue and turquoise modules were identified significantly correlated (*P* < 0.01) with virus amplification. Six viral proteins of SARS-CoV-2 were found to be clustered in the blue module, indicating that those viral proteins play an important role in the amplification of SARS-CoV-2 in host cells. The GENIE3 results showed that in comparison with other viral proteins, ORF3a had the highest weight for the regulation of host proteins, indicating that ORF3a plays an important role in the replication of SARS-CoV-2 in host cells. Previous studies on SARS-CoV have reported a prominent reduction of the virus following ORF3a deletion [24], and ORF3a was proposed to utilize multiple pathways to promote apoptosis in virus-infected cells [25]. Further analysis of GO enrichment showed that the main function of ORF3a was related to ER organization and macroautophagy, which was consistent with the results reported by Freundt et al. [26]. The results obtained for other viral proteins were also supported by several previous studies. For example, PP1A was reported to play a role in the initial induction of autophagosomes from the ER and in signaling pathways modulating the survival of host cells [27–29]. The latest study reported that ORF6 led to disordered metabolic molecular markers in the blood of SARS-CoV-2 patients [30]. As shown in (Table 1), the top ten proteins coexpressed with the viral proteins in the blue module demonstrated that TMBIM6 showed prominent coexpression with all detectable viral proteins, indicating that TMBIM6 is a key element influenced by the infection of SARS-CoV-2 in the cells. To determine whether the regulation of TMBIM6 expression occurred at the posttranscriptional level, we performed systematic studies on the expression of TMBIM6 with open access transcriptome, translatome and proteome data. Interestingly, we found that there was no significant difference in the expression of TMBIM6 at the transcriptional level; in contrast, there was a significant decrease in the expression of TMBIM6 at posttranscriptional levels, including both the translatome and proteome levels (Figure 4B–4D).

To certify that this phenomenon was induced by replication of SARS-CoV-2, we performed an analysis of multiomics data from mock-infected Caco-2 cells and found that the mock failed to downregulate the expression of TMBIM6 at both the mRNA and protein levels (Supplementary Figure 4A–4C). The idea that TMBIM6 is influenced by SARS-CoV-2 at the posttranscriptional level is also supported by several lines of evidence. For example, TMBIM6 expression was not significantly different in autopsied lung tissues from deceased COVID-19 patients at the transcriptional level. Consistently, the results obtained with the multiomics data from SARS-CoV-2-, IAV- or RSV-invaded cells demonstrated that TMBIM6 protein was specifically downregulated by SARS-CoV-2 but not other viruses at the posttranscriptional level. In comparison with other respiratory viruses, SARS-CoV-2 was found to have a high infection rate. We speculate that SARS-CoV-2 has specific targets that are different from those of other respiratory viruses and that TMBIM6 is an important potential target influenced by SARS-CoV-2 in host cells.

To date, the mechanism by which SARS-CoV-2 regulates the translation and synthesis of host proteins has not been clearly defined. For the decreased protein expression of TMBIM6 in the cells infected with SARS-CoV-2, we considered two aspects to provide a reasonable explanation. On the one hand, host protein translation is restrained by the competition of viral proteins, resulting in decreased expression of host proteins. For example, Bojkova et al. reported that viral proteins compete with host proteins for efficient translation [10]; Thoms et al. reported that nonstructural protein 1 (nsp1), a major virulence factor of SARS-CoV-2, suppresses host protein expression by ribosome association [31]. Additional analysis of SARS-CoV-2 also supports this idea; Alonso et al. reported that the preferred codons of SARS-CoV-2 have poor representation of G and C nucleotides in the third position, which could lead to an imbalance in the tRNA pools of the infected cells, with serious implications for host protein synthesis [32]. Based on these findings and our results, we speculate that the translation of six viral proteins leads to inefficient translation of host protein TMBIM6. Similar to nsp1, the six viral proteins may also inhibit the translation of TMBIM6 by binding to ribosomes. The consumption of specific tRNAs for the replication of the six viral proteins may restrict the synthesis of host proteins. On the other hand, the intracellular mechanisms controlling protein synthesis in host cells are hijacked by SARS-CoV-2 for massive synthesis of viral proteins, which leads to the accumulation of more misfolded and unfolded proteins, including TMBIM6, in the ER cavities. Subsequently, the unfolded protein response (UPR) occurs, and the host cells may stop protein translation or degradation of misfolded proteins to restore homeostasis and cell function.

**Figure 6:**
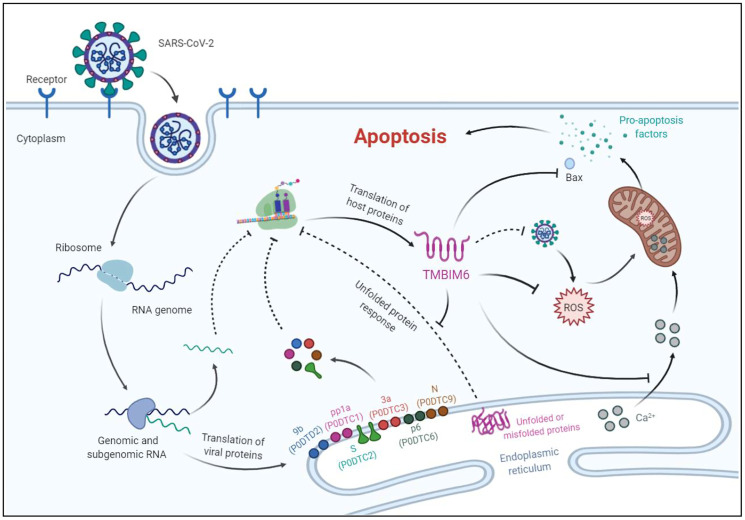
**A proposed working model for the apoptosis of host cells infected with SARS-CoV-2.** Possible mechanisms of host cell apoptosis induced by SARS-CoV-2 through translational inhibition of TMBIM6. TMBIM6 is a multifunctional protein that can inhibit the activation of BAX, ROS production and Ca2^+^ release from the ER. Upon SARS-CoV-2 infection, downregulated TMBIM6 promotes the release of Ca2^+^ and ROS accumulation in mitochondria, which leads to the release of proapoptotic factors. In addition, downregulation of TMBIM6 relieves its inhibition of BAX activation, thus leading to host cell apoptosis.

TMBIM6 is a multifunctional protein with the ability to inhibit the function of viral genes, attenuate UPR and ER stress [33], regulate calcium release [34], reduce the production of reactive oxygen species (ROS) [35, 36], activate Bcl-2 and inhibit BAX [37]. Previous studies demonstrated that the TMBIM6 protein is involved in the regulation of cellular responses to influenza virus infection by inhibiting the function of viral genes. For example, overexpression of TMBIM6 in MDCK cells impaired influenza virus production, viral propagation, and the synthesis of viral genes; MDCK cells overexpressing TMBIM6 were more resistant to influenza virus infection and virus-induced cell death [38]. Based on the results of bioinformatics analysis, we speculate that the decrease in the protein expression of TMBIM6 promotes continuous activation of the UPR. Under pathophysiological conditions, the UPR activates corresponding signaling pathways to produce more molecular chaperones for protein folding, and it is also possible that the UPR is utilized by SARS-CoV-2 to increase the assembly capacity of the ER to meet the protein synthesis needs of viral replication, thus promoting new virion assembly and the release of SARS-CoV-2 from infected cells. As described in the working model which was created by BioRender in (Figure 6), the balance of Ca2^+^ in the ER is disrupted, and ROS induced by SARS-CoV-2 cannot be controlled effectively due to the lack of the TMBIM6 protein, leading to an increase in Ca2^+^ concentrations and accumulation of ROS in mitochondria. Consequently, the increase in mitochondrial permeability causes the release of proapoptotic factors and the activation of BAX, leading to host cell apoptosis. Other lines of evidence also support the idea that TMBIM6 plays an important role during SARS-CoV-2 infection. The data of Alex et al. indicated that SARS-CoV S protein-induced membrane fusion is dependent on calcium [39] and Marco et al. reported that calcium channel blockers inhibited SARS-CoV-2 infectivity [40]. So, we infer that the decrease in the TMBIM6 expression leads to the disorder of Ca^2+^ concentration, which promotes the infection of SARS-CoV-2. Although the prominent clinical symptoms of SARS-CoV-2 are lung related [41], the impact of SARS-CoV-2 on other tissues has also gradually been uncovered; several recent studies have revealed that SARS-CoV-2 infects various organs, such as the brain [42], heart [43], gastrointestinal tract [44] and kidney [45], in addition to the lung. Consistently, in the HPA database, the TMBIM6 protein was highly expressed in most human tissues, including the brain, heart muscle, lung, bronchus, small intestine, kidney, etc. Recently, it was reported that cancer patients infected with SARS-CoV-2 have a higher risk of severe events and a poorer prognosis than those without cancer [46]. Through analysis of the GEPIA2 database [47], we found that the TMBIM6 transcription level was even higher in most cancers. These results suggest that SARS-CoV-2 may damage various organs by downregulating the expression of TMBIM6 protein, and may also affect the prognosis of cancer patients.

In summary, by performing bioinformatics analysis of multiomics data, we identified TMBIM6 as a potential viral regulatory protein in SARS-CoV-2-infected cells. However, our study has some limitations, such as a lack of experimental evidence, especially *in vivo* results to confirm our findings, because the high-grade BSL facility at our institution was inaccessible. Chemical compound screening and drug design targeting TMBIM6 might provide a novel clinical approach for the treatment of COVID-19 in the future.

## MATERIALS AND METHODS

### Differential gene and protein expression analysis

Differential gene expression analysis was performed with Limma-voom (version 3.42.0) [48] to compare gene expression in SARS-CoV-2-infected cells at different times using the transcriptome data of Emanuel et al. [11]. For the small sample number [48], we considered genes with a *P* value, rather than Q value, < 0.05 and an absolute value of log_2_ (fold change) > 1 as DEGs; conversely, genes with a *P* value ≥ 0.05 or an absolute value of log_2_ (fold change) ≤ 1 were considered non-differentially expressed genes (NDEGs). Also, through the transcriptome data of autopsied lungs, we utilized Limma-voom to calculate statistic difference and conform the expression of TMBIM6 at the transcriptional level. As for the transcriptome data of different virus-infected A549 cells, we utilized Deseq2 to calculate the statistic difference of the expression of TMBIM6 according to the original author. Similarly, using the proteome and translatome data of Bojkova et al. [10], differentially expressed proteins (DEPs) and non-differentially expressed proteins (NDEPs) were defined by statistical analysis with two-sided unpaired student’s t test with equal variance assumed according to the original author. *P* value < 0.05 and an absolute value of log_2_ (fold change) > 1 were regarded as the cut-off criteria.

### Gene set enrichment analysis

To gain insight into the activation of signaling pathways in SARS-CoV-2-infected cells, we performed GSEA on the proteome data with the “clusterProfiler” [49] and “GSEABase” [50] R packages. In details, gene set size > 5 and < 500, and *P* value < 0.05 were regarded as the cut-off criteria. Protein expression at 2, 4, 10 and 24 h was compared between SARS-CoV-2-infected cells and mock-infected cells, respectively.

### Protein coexpression network analysis

The R package WGCNA [51] was applied to find clinical trait-related modules and hub proteins within those modules using the proteome data of Bojkova et al. According to the topological overlap matrix (TOM)-based dissimilarity measure, proteins were divided into different modules. Here, we set the soft-thresholding power as 8 (scale-free R^2^ = 0.85), cut height as 0.25, minimal module size as 50 and threshold as 0.2 to identify key modules. Modules with the highest and lowest correlations with virus amplification over time were selected to explore their biological functions through ClueGO analysis. Hub proteins were defined as those coexpressed with virus proteins in the selected modules.

### Functional enrichment analysis

To further understand the mechanism of cellular processing, we conducted GO enrichment analysis using the Cytoscape [52] software “ClueGO” [53], “WEB-based GEne SeT Analysis Toolkit” (WebGestalt) [54] and R package “clusterProfiler” [49]. GO terms with a *P* value < 0.05 were considered statistically significant and visualized with the “ClueGO” and “ggplot2” R packages [55, 56].

### Gene regulatory network analysis

GENIE3 [57, 58] is an algorithm to infer gene regulatory networks from protein expression data. As for weight, the official document states that the choice of weight is like this: “The weights of the links returned by GENIE3() do not have any statistical meaning and only provide a way to rank the regulatory links. There is therefore no standard threshold value, and caution must be taken when choosing one.” Moreover, in the example of official documents, weight > 0.1 is selected as the screening standard. But for our data, there are too many proteins screened out by this standard, so we want to screen the appropriate number according to a certain proportion. We try a variety of screening methods and finally take the top 0.2%. According to this standard, we get the relationship between three virus proteins and host proteins. There are six virus proteins interacting with host proteins, and we just got 3 virus proteins at the top 0.2% (77 proteins). The weight between the other three virus proteins and host proteins is low. It is difficult to screen according to a certain proportion, so we selected the top 30 host proteins regulated separately by the remaining three virus proteins to do enrichment analysis.

### Analysis of protein expression in human tissues

The HPA [59] aims to map all human proteins in cells, tissues and organs by integrating various omics technologies, including antibody-based imaging, mass spectrometry-based proteomics, transcriptomics and systems biology. The HPA database was used to search the TMBIM6 protein expression in most tissues.

### Analysis of mRNA expression

Following a standard processing pipeline, GEPIA2 [47] was used to analyze RNA sequencing data for 9,736 tumors and 8,587 normal samples from TCGA and GTEx projects. The TMBIM6 expression profile at the transcriptional level was characterized with GEPIA2 across all tumor samples and paired normal tissues.

### Data and materials availability

All data associated with this study are presented in the paper.

## Supplementary Material

Supplementary Figures

Supplementary Tables
