# Supplementary material for: TMBIM6, a potential virus target protein identified by integrated multiomics data analysis in SARS-CoV-2-infected host cells
Source: Aging (Albany NY). 2021 Mar 19;13(7):9160–85. doi: 10.18632/aging.202718 (PMC8064151; doi:10.18632/aging.202718)
Supplement: Supplementary Figures [file aging-13-202718-s001.pdf]

SUPPLEMENTARY FIGURES

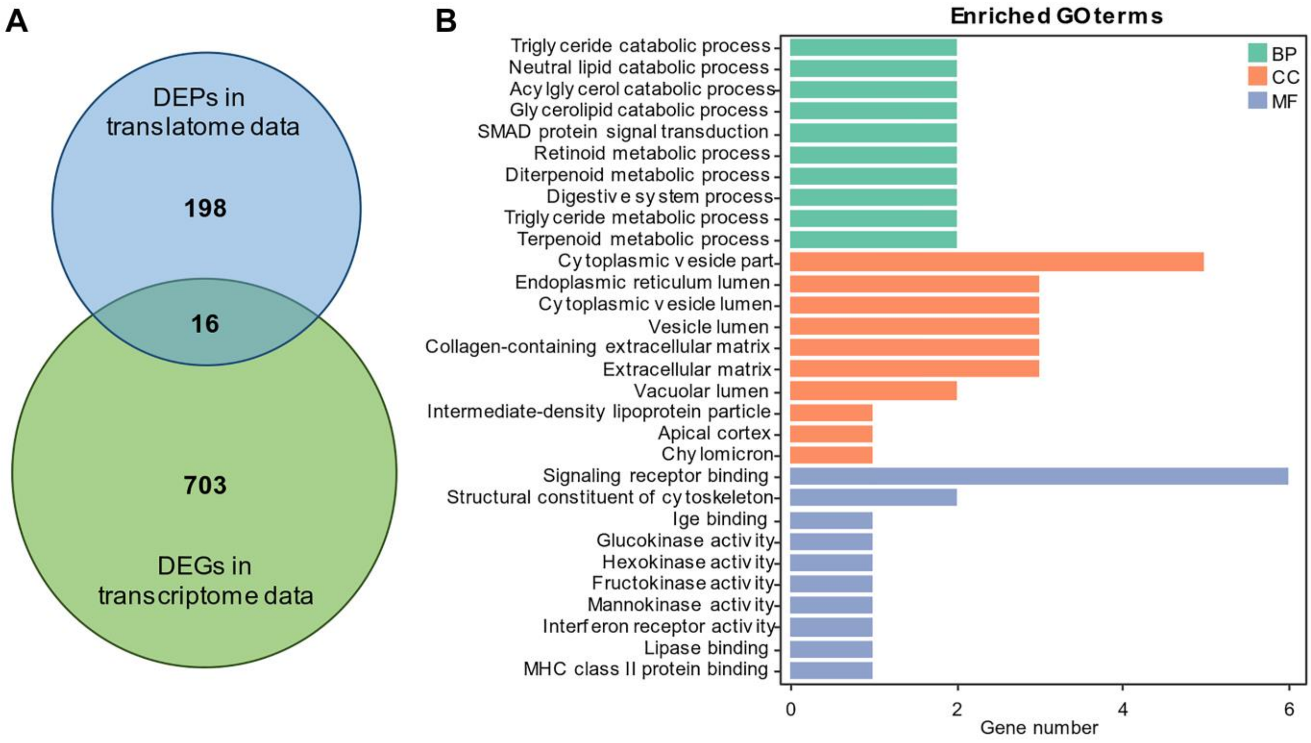

Supplementary Figure 1. GO enrichment analysis of differentially expressed proteins shared by the translatome and transcriptome. (**A**) Venn diagram showing the overlap of proteins encoded by the differentially expressed genes (DEGs) in the transcriptional data and differentially expressed proteins (DEPs) in the translational data. (**B**) GO term enrichment analysis for the 16 proteins shared by DEG-encoded proteins and DEPs with *P* value < 0.05. (BP: Biological Process; CC: Cellular Component; MF: Molecular Function).

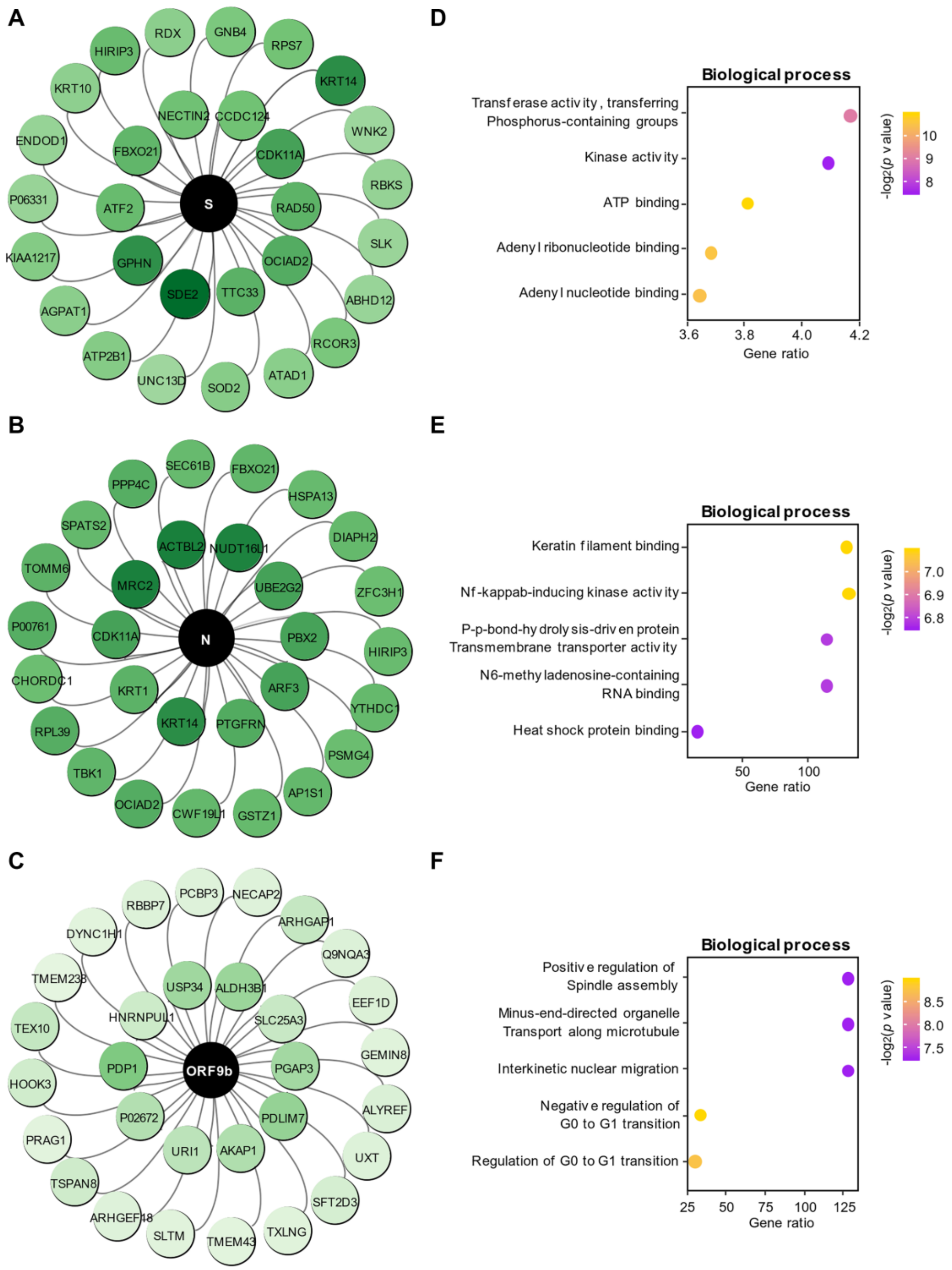

Supplementary Figure 2. Functional prediction for the other three viral proteins in the blue module. (**A**–**C**) The paired associations of the other three viral proteins, S (A), N (B) and ORF9b (C), and the top 30 high-weight host proteins for regulation predicted by GENIE3. The color depth represents the weight of the pairing between viral proteins and each host protein. (**D**–**F**) Enrichment analysis of biological processes of host proteins regulated by the viral proteins S (D), N (E) and ORF9b (F).

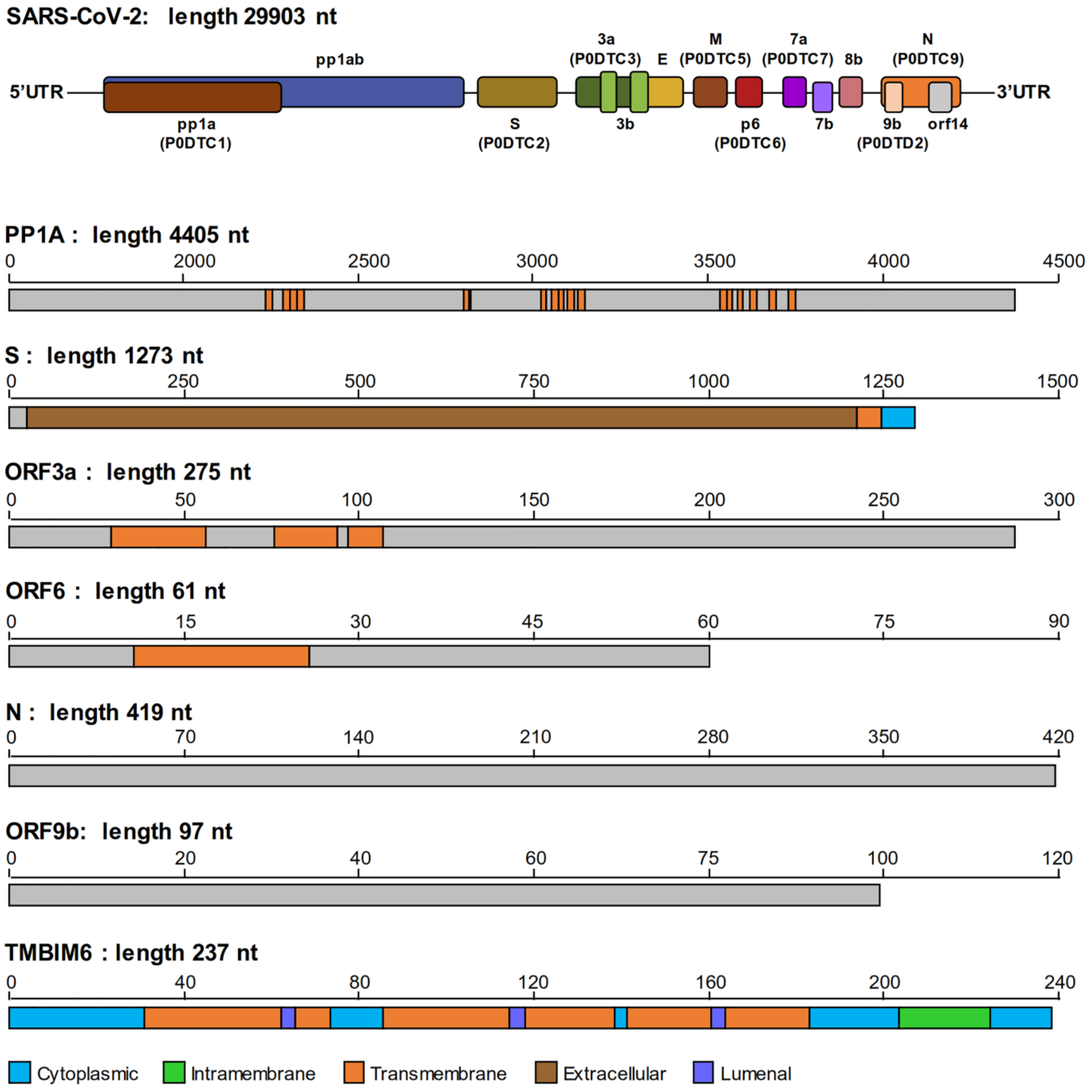

Supplementary Figure 3. Sketch of the functional domains of TMBIM6 and the six viral proteins. The genome composition of SARS-CoV-2 and a schematic diagram of the protein domains of TMBIM6 and the six viral proteins. Specific domains are shown in distinct colors. Information on the domains of TMBIM6, PP1A, S and ORF3a was collected from the UniProt Consortium, and the others were predicted with PredictProtein (PP).

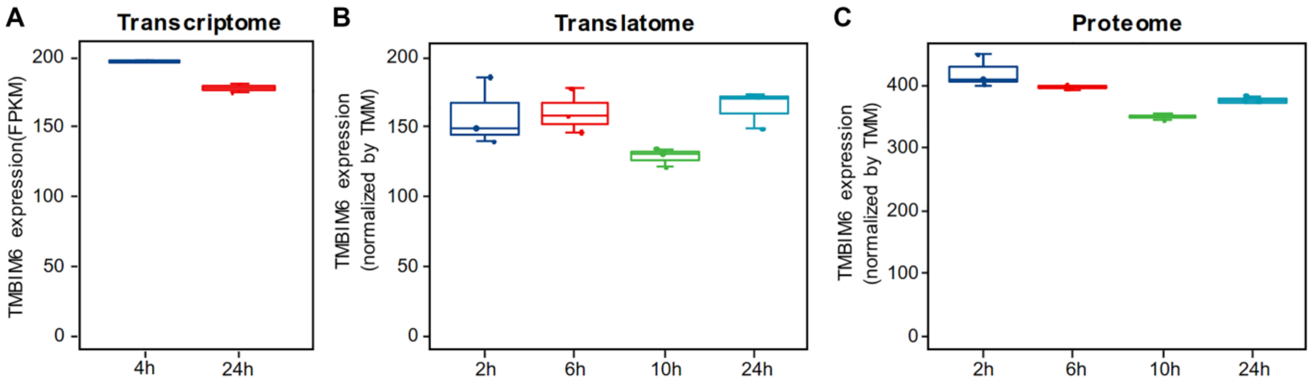

Supplementary Figure 4. Expression of TMBIM6 in the control group. The expression of TMBIM6 at different times in mock-infected cells from multiomics data. (**A**) The gene expression of TMBIM6 in the transcriptome in normal cells. (P value > 0.05, comparison to 4 h, n = 2). The P value was calculated with Limma-voom R package following the method reported by Ritchie ME et al. [48]. (**B**) Nascent peptide synthesis of TMBIM6 in the translatome of mock-infected cells. (n = 3, P value > 0.05, comparison to 2 h) (**C**) The protein expression of TMBIM6 in the proteome of mock-infected cells. (n = 3, P value > 0.05, comparison to 2 h).

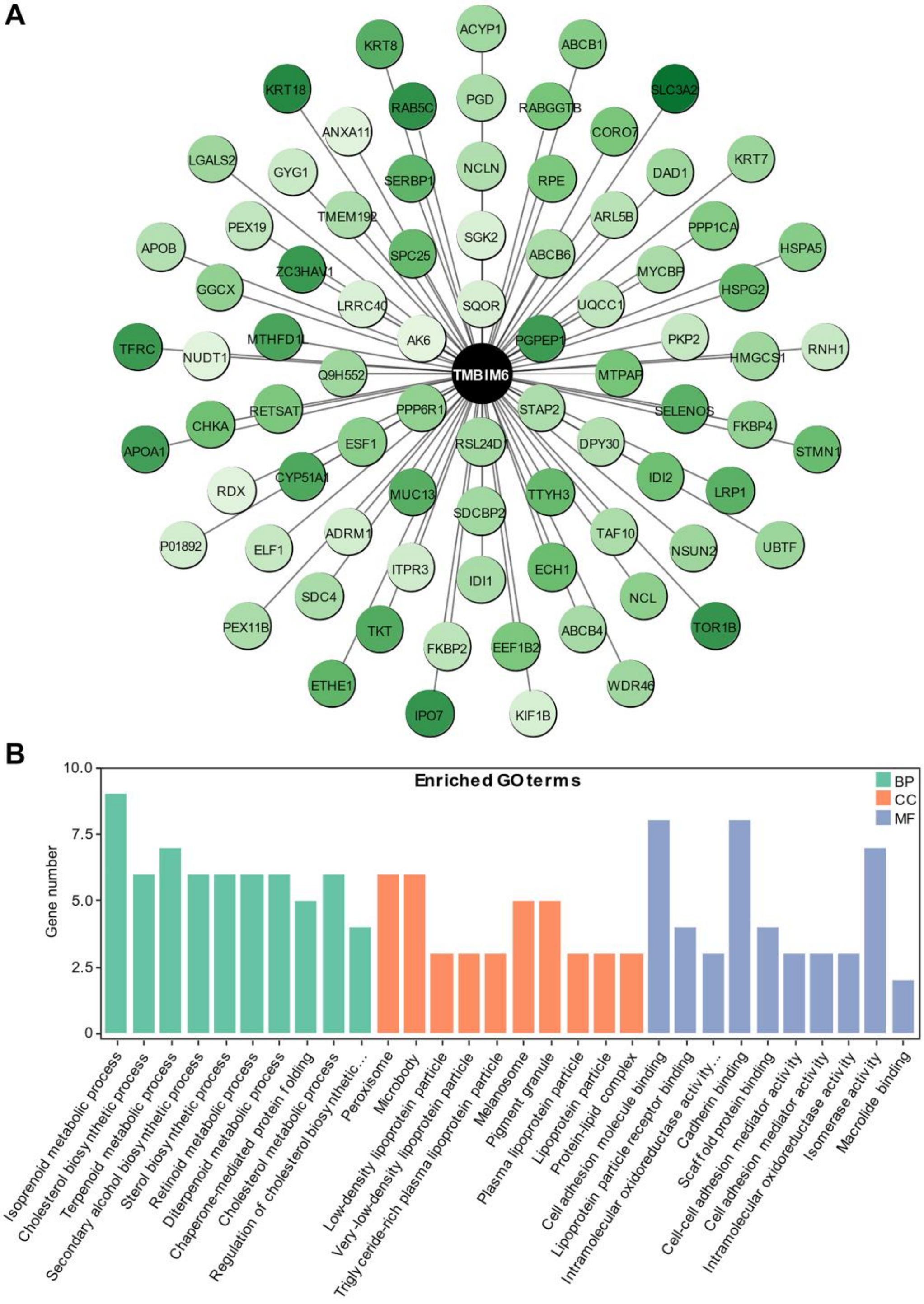

Supplementary Figure 5. Enrichment analysis of the proteins coexpressed with TMBIM6. (**A**) Association of TMBIM6 with the coexpressed proteins in the blue module. The color depth represents the weight of the pairing between TMBIM6 and each host protein. (**B**) GO term enrichment analysis of the proteins coexpressed with TMBIM6 (*P* value < 0.05). (BP: Biological Process; CC: Cellular Component; MF: Molecular Function).
