# Supplementary material for: TMBIM6, a potential virus target protein identified by integrated multiomics data analysis in SARS-CoV-2-infected host cells
Source: Aging (Albany NY). 2021 Mar 19;13(7):9160–85. doi: 10.18632/aging.202718 (PMC8064151; doi:10.18632/aging.202718)
Supplement: Supplementary Tables [file aging-13-202718-s002.pdf]

SUPPLEMENTARY TABLES

Supplementary Table 1. A549 proteins dysregulated by infection by indicated influenza strains.

Protein	Fold-Change Compared to Sham-Infected H1N1 Viruses				
	PR8	RV733	pdm09	H5N1	H7N9
			Up-Regulated Proteins		
ISG15	15.9	1.03	0.96	1.33	1.1
OAS1	5.06	0.99	1.08	0.97	0.96
CCL5	3.11	1.06	1.09	4.18	1.62
STAT1	3.09	1.26	0.85	1.02	1
B2M	2.72	0.97	1.11	1.12	0.99
APOL1	2.68	0.98	0.99	0.99	0.92
CD274	2.09	1.06	1.05	1.27	1.13
CTSS	2	0.99	1.05	0.79	0.59
SERPINE1	1.99	1	1.1	0.89	0.7
IFNL1	1.97	1	0.99	1.52	1.21
F2	1.84	1.03	1	0.97	0.89
PLAUR	1.8	0.98	1.05	1.05	0.85
MDK	1.8	1.01	0.97	1.02	0.93
CFB	1.75	1.04	1.03	1.03	0.96
THPO	1.01	1.58	1.01	1	1.1
L1CAM	1.21	1.28	1.83	1.57	1.47
CXCL8	1.26	1	0.99	2.26	2.3
CD207	1.1	1.05	1.06	1.61	1.38
F9	2.21	1.03	1.14	1.53	1.29
			Down-regulated proteins		
PPID	0.66	1.15	0.95	0.97	1.03
TGM3	0.88	0.61	1.21	0.75	0.9
PGAM1	1.05	0.83	0.39	0.54	0.5
MDH1	1.01	1.02	0.48	0.59	0.83
LDHB	0.97	1.19	0.61	0.84	1.02
ENO1	0.91	0.96	0.63	0.77	0.82
PCSK9	0.8	0.92	1.07	0.17	0.11
DKK1	0.85	0.87	0.92	0.19	0.17
DKK4	0.88	0.91	0.97	0.25	0.22
APP	0.93	0.94	0.99	0.27	0.12
SPINT2	0.93	0.87	0.95	0.37	0.19
TNFRSF4	1.01	0.53	0.56	0.39	0.54
IGFBP4	1.28	0.95	1.06	0.41	0.21
PGD	0.98	1.12	0.8	0.44	0.66
FN1	1.12	0.94	0.95	0.47	0.4
TGFBI	0.87	0.95	0.95	0.48	0.39
SGTA	0.93	1.01	0.65	0.49	0.8
GAPDH	0.97	1	0.7	0.5	0.83
FSTL3	1.49	0.92	1.04	0.5	0.33
FN1	1.12	0.95	0.94	0.53	0.47

CTSA	0.75	0.94	1.04	0.53	0.35
MICB	0.88	0.93	1	0.57	0.6
NOTCH3	1.06	0.9	0.95	0.59	0.47
PKM2	0.8	1.1	1.38	0.59	0.81
LRIG3	1.5	0.87	0.92	0.59	0.63
MFGE8	0.91	0.94	1.05	0.63	0.43
PEX5	0.98	0.71	0.73	0.63	0.75
WNK3	1.1	0.93	0.86	0.63	0.77
TNFRSF21	0.96	0.92	0.96	0.63	0.43
SFRP1	1.27	1	0.98	0.64	0.45
TNFRSF1A	0.74	0.97	1.09	0.64	0.46
FSTL1	1.28	0.92	1.02	0.65	0.44
IGFBP7	1.06	0.96	1	0.65	0.53
NRP1	1.09	0.94	0.89	0.65	0.53
CSF3R	1.03	0.75	0.83	0.66	0.75
C3	1.12	0.96	1	0.66	0.61
CFH	1.54	1	0.96	0.66	0.52
STC1	1.19	0.89	1.05	0.69	0.37
FGFR1	1.02	0.94	1.02	0.69	0.47
CTSV	0.83	0.98	1.08	0.74	0.52
CST3	1.39	0.94	1.1	0.86	0.55
PLXNB2	1.05	1.02	1.14	0.71	0.58
LGALS8	1.18	0.92	0.92	0.78	0.59
NRG1	1.21	1.03	1.24	0.94	0.6
GNS	0.95	1.04	1.09	0.75	0.6
MICA	0.85	1.04	1.18	0.68	0.61
LAMA1<br>LAMB1<br>LAMC1	0.76	1.09	1.14	0.79	0.62
THBS1	1.05	1.01	1.04	0.68	0.62
TIMP2	0.97	0.93	1.03	0.85	0.62
MMP7	0.93	0.99	1.03	0.93	0.63
LCN2	0.78	0.96	1.03	0.87	0.63
GRN	1.04	0.96	1.04	0.67	0.63
TFPI	0.8	1.03	1.03	1.1	0.63
GFRA1	0.77	0.97	0.99	0.75	0.63
MET	1.23	1.03	1.11	0.89	0.64
KIR2DL4	0.92	1.01	0.92	0.77	0.65
LGALS3BP	1.49	0.97	1.01	0.83	0.65

Values represent protein fold-changes compared to mock-infected. Proteins sorted first by upregulation and from left-most virus column to right-most; then sorted by downregulation from left to right.

Supplementary Table 2. Differentially airway expressed proteins compared between children with and without RSV infection.

Protein	Log_{10} mean expression in RSV-negative group	Log_{10} mean expression in RSV-positive group	*P*.value	FDR
CAT	5.114667484	5.301170781	1.28E-08	6.15E-06
HP	4.943199091	5.132428762	2.00E-08	7.20E-06
MNDA	5.017074398	5.433427343	4.17E-08	1.15E-05
RAP1B	4.949246377	5.183158396	4.77E-08	1.15E-05
MYH9	5.518510344	5.823424089	7.16E-08	1.48E-05
MMP9	4.708812819	5.100992611	1.29E-07	2.07E-05
EMC7	4.490219365	4.717223378	1.71E-07	2.33E-05
ITGAM	5.292602525	5.593034483	1.92E-07	2.33E-05
MGAM	4.419582833	4.778247554	6.23E-07	6.42E-05
APMAP	5.074535297	5.32660057	7.62E-07	7.32E-05
MPO	6.140891698	6.318606892	2.25E-06	0.000170608
BPI	5.097592111	5.404436702	3.30E-06	0.000207032
AZU1	4.886635066	5.161970143	7.38E-06	0.000364581
MYO1F	4.511891333	5.01680507	7.58E-06	0.000364581
SERPINA3	5.055420395	5.292196687	1.07E-05	0.000466571
HSP90AB1	5.139026935	5.341131197	1.10E-05	0.000466571
ANXA6	5.655306917	5.853015652	1.25E-05	0.000491166
PADI4	4.589404054	4.991695909	1.40E-05	0.000530222
EPX	4.604312816	5.590438279	1.85E-05	0.000679374
GCA	4.863981185	5.270911516	1.93E-05	0.000679374
MYDGF	4.339989914	4.756786345	1.98E-05	0.000679374
CANX	5.048917062	5.2431655	2.10E-05	0.000681136
SRSF3	4.218063025	4.437589361	2.44E-05	0.000765642
LCN2	5.326103066	5.503063103	3.96E-05	0.001119608
HP1BP3	4.293497481	4.539154806	4.09E-05	0.001134989
ELANE	4.93978268	5.127932395	5.37E-05	0.001389106
RAB10	4.238116644	4.440085185	6.31E-05	0.001459194
LCP1	5.257523438	5.464338395	6.56E-05	0.001478903
CPNE1	4.726319498	4.926530454	6.84E-05	0.001516836
SH3GLB1	4.297194109	4.602835986	0.000127054	0.002359089
KIF26B	5.257184823	5.456586151	0.00015695	0.002760015
CTSS	3.911710292	4.087836721	0.000159594	0.002772701
ACOX1	4.419834462	4.698403156	0.000168715	0.002868469
PSME2	4.264533049	4.521832593	0.000238025	0.003813696
ITGB2	5.05813684	5.266248026	0.000245421	0.003879711
HNRNPAB	4.438715726	4.649400188	0.000248558	0.003879711
GRHPR	4.266930668	4.448939488	0.000250217	0.003879711
PRTN3	4.807077714	5.067267571	0.000270166	0.004058111
ALOX5	4.541069623	5.083127355	0.000355657	0.005128569
DCD	4.394689684	4.690283061	0.000384398	0.005488132
NDUFA11	4.164125357	4.48487794	0.0005119	0.006963769
DYSF	4.666788893	4.996987759	0.000527755	0.007112359

MYH14	4.638495555	4.866330565	0.000572568	0.007543538
S100A12	4.941173281	5.304467151	0.000654068	0.008168783
TMED2	4.271798725	4.485082471	0.000713832	0.00857788
MX1	4.881645757	5.224818513	0.001023358	0.011012558
NDUFA12	4.244562117	4.587710606	0.001158382	0.012058949
CAMP	4.721340789	4.916958008	0.001269447	0.012800992
CKB	4.023948761	4.257760936	0.001290731	0.012903917
CYBB	4.710832198	4.988796789	0.001421212	0.013074125
PLBD1	4.537548032	4.768849146	0.001442147	0.013074125
ORM1	4.819126792	5.02006486	0.001490856	0.013074125
PPIC	3.885470221	4.153959038	0.001495999	0.013074125
TCEB2	4.138376822	4.333947036	0.001651799	0.013880687
PCMT1	4.247477859	4.439112435	0.001955052	0.015575607
TLN1	4.679858981	4.872637538	0.002140904	0.016788022
TRAP1	4.294250303	4.574332382	0.002494947	0.019035414
ARPC5	4.272058349	4.57568884	0.002800627	0.020710274
SERPINB10	4.224020012	4.475496364	0.003130255	0.022456852
PTPRC	4.830039126	5.076758062	0.003299715	0.023324454
RETN	4.312720031	4.543402821	0.00348406	0.024153914
EHD1	4.231194433	4.425856813	0.004084851	0.027072375
FTH1	4.704243637	4.901463071	0.004183767	0.02742425
ANPEP	4.201451437	4.441455196	0.004184005	0.02742425
RBM39	3.96989748	4.233939707	0.004779836	0.029967494
GNS	4.23532213	4.587026036	0.005160762	0.031802644
PLA2G4B	3.319511058	3.538414019	0.005824789	0.034997275
OLFM4	4.88179311	5.225560007	0.00641208	0.037894343
GSTO1	4.329614511	4.507592378	0.006545645	0.038213845
HPRT1	4.02449349	4.246696767	0.00698618	0.039818463
MAPRE1	4.341162146	4.520932463	0.007341398	0.041204574
TOMM40	4.268530324	4.467013054	0.008485019	0.04565447
CALR	4.839972595	5.081679102	0.009534153	0.049993634
RPL10	5.038303341	4.825068869	1.52E-09	2.20E-06
CES2	4.739516838	4.539885993	8.27E-09	5.96E-06
ILF3	4.480517964	4.078762258	8.69E-08	1.57E-05
PSMD11	4.563260965	4.311806318	4.97E-07	5.51E-05
HNRNPR	4.818464478	4.486926813	1.11E-06	9.97E-05
CAMK2D	4.64026272	4.323096185	1.43E-06	0.00012137
DNAJB1	4.410924636	4.080041455	3.16E-06	0.000207032
KRT19	4.390867068	4.073041713	3.90E-06	0.000234244
RPS25	4.494494689	4.1554186	5.95E-06	0.000317985
NDUFA5	4.252835291	3.800853144	7.36E-06	0.000364581
CORO1B	4.311809276	3.840996573	1.26E-05	0.000491166
BAZ1B	4.331136327	4.071409626	2.58E-05	0.000792894
KLK10	4.610027666	4.350204027	3.07E-05	0.000903416
PRPF19	4.276525779	4.099943658	4.18E-05	0.001137632
RPL17	4.588634204	4.389761832	5.56E-05	0.001389106
AGR3	4.641451574	4.197310764	5.75E-05	0.001389653

IPO9	4.547905831	4.12918725	6.38E-05	0.001459194
RBMX	4.538922051	4.362244876	0.000104859	0.002071318
CSNK2B	4.472511277	4.272629227	0.000117422	0.002257634
RPS6	5.136831181	4.949633885	0.000138693	0.002531581
PGRMC2	4.377971929	4.143431274	0.000149387	0.002692698
SUGP2	4.1219491	3.919151077	0.000176012	0.002951263
RUVBL2	4.641147664	4.39312991	0.000197676	0.003276429
RPL29	4.456132726	4.228694489	0.000388869	0.005497545
PSMA3	4.375285194	4.160471533	0.000575443	0.007543538
KHDRBS1	4.533721407	4.324410349	0.000594524	0.007723456
ARCN1	4.543623773	4.33631855	0.00077428	0.009189714
ABHD11	4.608569859	4.25672282	0.00078057	0.009189714
NDUFA2	4.551278157	4.340961981	0.000866266	0.009759027
RPS16	4.692821753	4.498170909	0.000999374	0.010835322
RPS14	4.473910141	4.197533028	0.001297551	0.012903917
SUMF2	4.3979048	4.144564048	0.001351827	0.013012456
NRAS	4.485524833	4.283700719	0.001362608	0.013012456
DCN	4.391247118	3.895792297	0.001395888	0.013074125
RPS29	4.434367987	4.230899371	0.001422306	0.013074125
PON2	4.126202606	3.889726328	0.001425059	0.013074125
DSC2	4.75835541	4.574709691	0.001521189	0.013214188
NAV3	4.160374551	3.975599012	0.001617661	0.013733623
VSNL1	4.528289229	4.285824839	0.001655671	0.013880687
SAP18	4.291686773	4.053549637	0.001802984	0.014772177
ARPC1A	4.56710576	4.379795065	0.001872618	0.015256022
TPPP3	4.844516599	4.626516636	0.001890712	0.015316895
TUBB6	4.331582035	4.111439776	0.002667592	0.019930921
BPIFB2	4.719890074	4.534950525	0.002936209	0.021383907
SPRR1B	4.599614991	4.414814995	0.003070743	0.022140054
PC	4.592197127	4.183016157	0.003386857	0.023593468
POLDIP2	4.568791291	4.218528593	0.003640583	0.024880191
ABHD10	4.227812135	4.040881793	0.004530498	0.028653414
NCF4	4.696260088	4.439957592	0.006964206	0.039818463
C2orf54	4.304826848	4.091812607	0.007882412	0.043632633
HADH	4.723118282	4.528480606	0.007927704	0.043632633
CMAS	4.053655261	3.795188414	0.007979096	0.043748506
AIMP2	4.321933547	4.144490597	0.008364574	0.045344796
DHX15	4.291289134	4.063572284	0.008698955	0.046631573
